# CUBN gene mutations may cause focal segmental glomerulosclerosis (FSGS) in children

**DOI:** 10.1186/s12882-021-02654-x

**Published:** 2022-01-03

**Authors:** Jing Yang, Yongli Xu, Linxia Deng, Luowen Zhou, Liru Qiu, Yu Zhang, Jianhua Zhou

**Affiliations:** 1 Department of Pediatrics, Tongji Hospital, Tongji Medical college, Huazhong University of Science and Technology, Jiefang Ave. No. 1095, Wuhan, 430030 China; 2 Department of Neurosurgery, Tongji Hospital, Tongji Medical college, Huazhong University of Science and Technology, Wuhan, China

**Keywords:** *CUBN* gene, Gene mutation, Focal segmental glomerulosclerosis, Proteinuria, Podocyte

## Abstract

**Background:**

Imerslund-Gräsbeck Syndrome (IGS) is mainly caused by CUBN gene biallelic mutations. Proteinuria accompanies IGS specific symptoms in about half of the patients, isolated proteinuria is rarely reported. Here we present 3 patients with isolated proteinuria and focal segmental glomerulosclerosis (FSGS) caused by CUBN gene biallelic pathogenic variants.

**Method:**

Whole exome sequencing was performed on three children with isolated proteinuria. *CUBN* gene biallelic pathogenic variants were found and then verified by sanger sequencing. Their clinical, pathological and molecular genetic characteristics were analyzed and correlated accordingly.

**Results:**

All three children presented with isolated proteinuria, no megaloblastic anemia. Their urine levels of β2 microglobulin were normal or slightly higher. Renal biopsies showed focal segmental glomerulosclerosis with mild glomerular mesangial hypercellularity, partial effacement of foot processes and podocyte microvillation. Two of them were found to carry compound heterozygous mutations and one homozygous mutation of *CUBN* gene. Totally four *CUBN* gene biallelic pathogenic variants were identified, including c.9287 T > C (p.L3096P), c.122 + 1G > A, c.7906C > T (p.R2636*), c.10233G > A (p.W3411*). Except for intron splice-site mutation, all other variants are located in highly conserved sites of CUB domain for binding to albumin.

**Conclusion:**

The results demonstrate that *CUBN* gene mutations may cause isolated proteinuria pathologically presented as FSGS. Our cases extend the spectrum of renal manifestation and genotype of *CUBN* gene mutations.

## Introduction

Imerslund-Gräsbeck Syndrome (IGS) is a rare autosomal recessive disease caused by mutations of *CUBN* or *AMN* gene [1, 2]. The clinical features of IGS include selective intestinal vitamin B12 malabsorption resulting in megaloblastic anemia, failure to thrive, recurrent infections, neurological damage, with or without proteinuria and normal renal function [3] . To date, only 48 different mutations in *CUBN* and 32 different variants in *AMN* have been reported in the “Human Gene Mutation Database” (HGMD) (http://www.hgmd.cf.ac.uk/ac/index.php).

*CUBN* gene encodes a large 460-kDa glycosylated extracellular protein called cubilin, which is composed of 27 CUB domains [4]. Cubilin expresses in both renal proximal tubular cells [4] and podocytes [5]. It contains eight Ca^2+^-binding epidermal growth factor repeats and a N-terminal region involved in trimerization of the protein [6]. The CUB domains of cubulin can act as ligand-binding sites for intrinsic factor-B12 complex [7], albumin [8, 9], vitamin carrier proteins, lipoproteins, other carriers, immune- and stress-related proteins and drugs [10]. Among them, the intrinsic factor-B12 complex binds to CUB domains 5–8 [11], but the binding sites for albumin remain unclear. Cubilin has no transmembrane domain in the structure, so it is dependent on additional factors for membrane anchoring and for endocytosis of the receptor-bound ligands [6]. As an endocytic receptor, cubilin mediates the uptake of proteins and protein-bound substances both in intestine and kidney [6]. The majority of filtrated albumin is reabsorbed through cubilin-mediated endocytosis in the proximal tubule, resulting in very low level of albumin in final urine.

In addition to Imerslund-Gräsbeck Syndrome, *CUBN* gene mutations have been reported in only a few cases with isolated proteinuria. The proteinuria is thought from tubular loss without glomerular involvement and the prognosis is benign [12–14]. In this paper, we report 3 patients with *CUBN* gene biallelic pathogenic variants presented as isolated proteinuria and FSGS in renal biopsy. Our cases extend the spectrum of renal manifestation and genotype of *CUBN* gene mutation.

## Materials and methods

### Patients

Patient 1 was a 11-year-old boy with asymptomatic persistent proteinuria of 2+ for more than one month with unknown causes. Patient 2 was a 6-year-old girl with foamy urine and proteinuria of 2+ for one year. Patient 3 was an 8-year-old boy with persistent proteinuria for more than one and half a year. All of them had no edema, hypertension, oliguria. Renal biopsy was performed and FSGS was reported in all of them.

### Clinical data collection, renal histopathological evaluation and follow-up study

All three cases were regularly followed up and evaluated through examination of blood biochemistry, serum immunoglobulin and complement, urine albumin/creatinine and β2 microglobulin levels. Tacrolimus was tried in these cases after renal biopsy, started at dosage of 0.05–0.1 mg/kg and maintained a stable trough concentration at 5-10 ng/ml for at least 3 months.

### Detection and analysis of *CUBN* gene variants by family trio whole exome sequencing

After the informed consent of the patients’ parents, we collected blood samples from the patients and their parents. Total DNA was extracted from peripheral blood using the QIAamp DNA Blood Kit (Qiagen) according to the kit standard protocol instruction. DNA were sheared with Covaris LE220 Sonicator (Covaris) to target of 150-200 bp average size. PCR amplification is performed using universal primers complementary to the adapter sequence to form a sequencing library. The sample genetic fingerprint was detected by Fluidigm Biomark using human specific SNP primers. The sequencing quality was determined by FastQC software performed on sequencing rawData. After data filtration, the clean reads were mapped to human reference genome (hg19) using Sentieon BWA software. Then, the mapped reads were detected to find SNV and InDel with Sentieon (the same algorithm with GATK) analysis, and annotated using ANNOVAR/VEP software. The pathogenic variants were screened by ClinVar, OMIM and HGMD. Functional prediction of missense mutation was determined by PolyPhen-2 and Sorting Intolerant from Tolerant (SIFT). Splice-cite prediction was determined by Human Splicing Finder (http: //www.umd.be/HSF/). The variants were evaluated by Clinic Sequence Analyzer (CSA) and classified according to “Standards and guidelines for the interpretation of sequence variants: a joint consensus recommendation of the American College of Medical Genetics and Genomics and the Association for Molecular Pathology” (published by ACMG, 2015), [15]. The variants were named according to the Human Genome Variation Society (HGVS) policy (https://www.hgvs.org/mutnomen). All the suspicious pathogenic variants were validated in patient and their parents by Sanger sequencing. The mutation sites were mapped in cubilin protein with DOG 2.0(Visualization of Protein Domain Structures) [16] and assessed for their conservation among different species using UCSC Genome Browser (http://genome.ucsc.edu/).

Sequences were submitted to the Swiss-Model server (https://swissmodel.expasy.org/) for modelling in automated mode, predicted effects of CUBN variants on protein structure were modelled using Swiss-PdbViewer [17].

## Results

### Clinical, renal pathological and follow-up findings

**Fig. 1:**
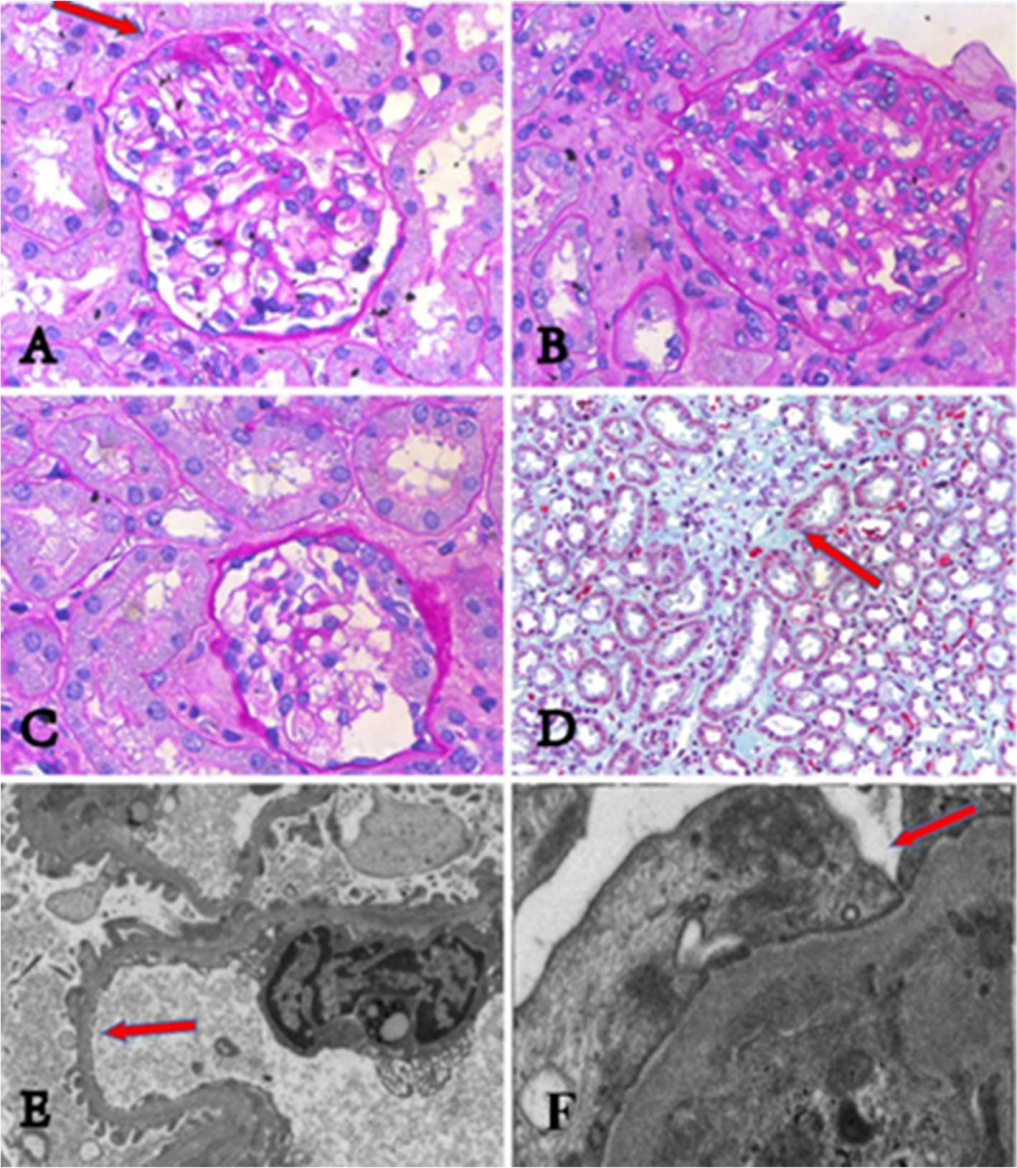
The renal pathologies of three patients. Kidney pathologies of three patients under light microscope are showing in Fig. 1 A-D. All of them have focal segmental glomerulosclerosis, mild mesangial cell proliferation, and interstitial fibrosis. (A) shows focal segmental glomerular sclerosis (pointed by red arrow) from patient 1. (B) and (C) show crescents in renal pathologies respectively from patient 1 and patient 2. (D) shows interstitial fibrosis (pointed by red arrow) from patient 1. podocyte pathologies of three patients under electron microscopy are showing in Fig. 1 E-F. All of them have effacement of foot processes and podocyte microvillation. (E) shows podocyte microvillation (pointed by red arrow) from patient 1. (F) shows effacement of foot processes (pointed by red arrow) from patient 2

**Fig. 2:**
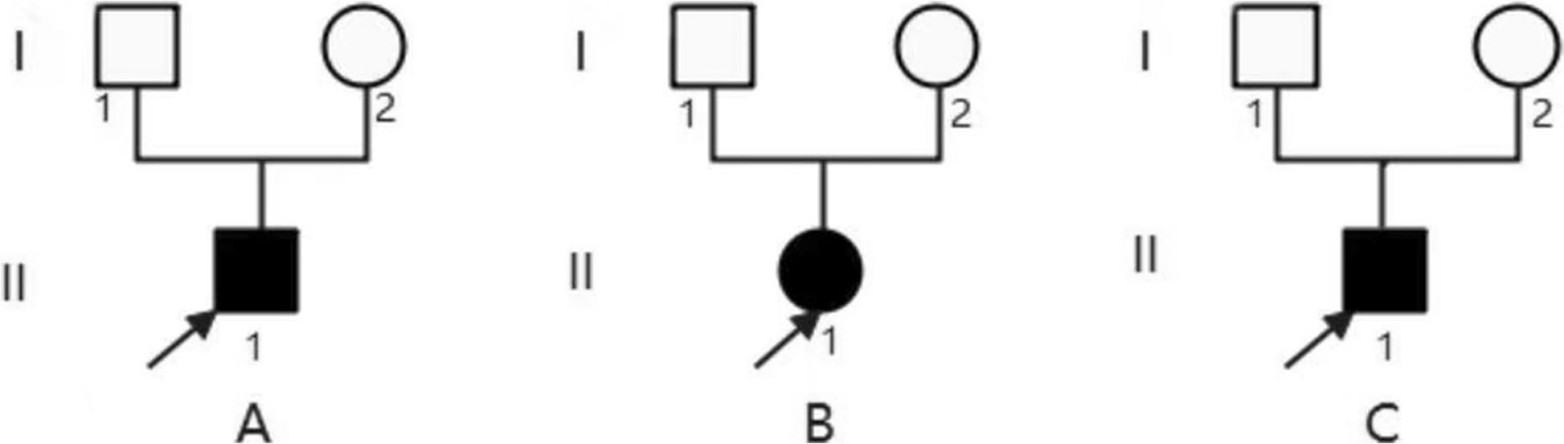
Family diagram of the three patients. A, B, C indicate patient 1, 2, 3 respectively

The clinical and renal pathological features were summarized in Table 1. All cases presented with proteinuria 2+ or over, urine β2 microglobulin was within the normal range in 2 cases and slightly increased in 1 case. Their urine protein electrophoresis showed albumin predominantly. Their hemoglobin (Hb) counts, serum IgG, IgA, IgM, C3, C4, and vitamin B12 levels were normal. All of them had normal renal functions, eGFR were 132, 165, 168 ml/min/1.73m^2^ respectively. Their renal ultrasonography showed no abnormalities. Renal biopsies showed segmental glomerulosclerosis (shown in Fig. 1 A), crescents (shown in Fig. 1 B, C), interstitial fibrosis (shown in Fig. 1 D). No immunoglobulin and complement deposited in the mesangial cells. Effacement of foot processes and podocyte microvillation were observed under electron microscopy (shown in Fig. 1 E, F). Neither of their parents has proteinuria, their family diagram of the three patients shown in Fig. 2. Urine albumin/creatinine values has decreased from 354μg/mg to 187μg/mg in patient 1, from 536μg/mg to 338μg/mg in patient 2, from 462μg/mg to 19.7μg/mg in patient 3 after administration of tacrolimus over three months.

**Table 1:** Clinical features of three patients

Patient	One	Two	Three
Age at onset (years)	10.7	5	6.5
Age at diagnosis (years)	11	6	8
Gender	Male	Female	Male
Edema	No	No	No
Megaloblastic anemia	No	No	No
proteinuria	2+	2+	2+
Urine protein/creatinine (ug/mg)	354	536	462
Urine β2-microglobulin	< 0.1 mg/L	< 0.1 mg/L	0.56 mg/L
Urinary protein electrophoresis	93.3% albumin	89.3% albumin	90% albumin
Hemoglobin(g/L)	115	116	130
Serum albumin	42.5	42.4	42.5
Egfr (ml/min/1.73m^2^)	132	165	168
LM			
Mesangial cell proliferation	Mild	Mild	Mild
Segmental sclerosis (proportion)	2/24	1/40	1/55
Interstitial fibrosis	Yes	Yes	Yes
Crescent (number)	1	1	0
IF	No deposits	No deposits	No deposits
EM			
Effacement of foot processes	Yes	Yes	Yes
Podocyte microvillation	Yes	Yes	Yes
Gene mutation			
Allele 1	c.9287 T > C, p. L3096P (Exon59)	c.9287 T > C, p. L3096P(Exon59)	c.10233G > A, p.W3411*(Exon64)
Allele 2	c.122 + 1G > A (Intron1)	c.7906C > T, p.R2636* (Exon51)	c.10233G > A p.W3411*(Exon64)

*eGFR* Estimated glomerular filtration rate; *LM* Light microscopy; *EM* Electron microscopy; *IF* Immunofluorescence

### Identification of *CUBN* gene mutation

**Fig. 3:**
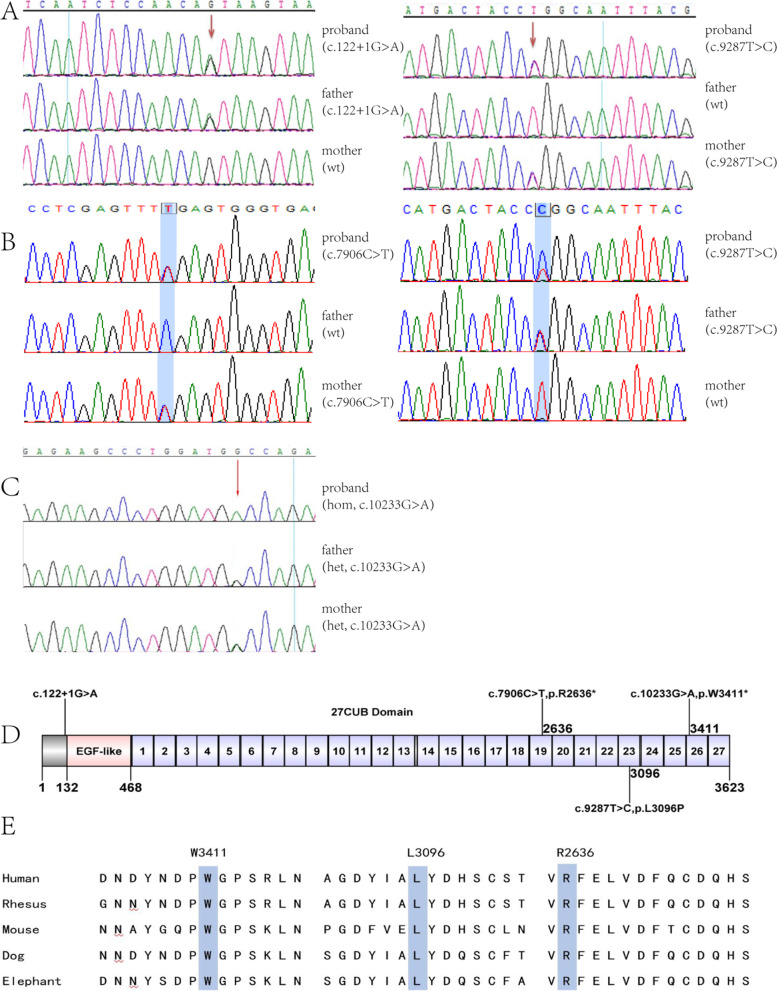
sanger sequencing confirmation and conservation analysis of CUBN gene mutations in three families. (A) Compound heterozygous mutation of CUBN gene in patient 1, his patients carry heterozygous CUBN gene mutation of c.122 + 1G > A and c.9287 T > C, respectively. (B) Compound heterozygous mutation of CUBN gene in patient 2, his patients carry heterozygous CUBN gene mutation of c.7906C > T and c.9287 T > C, respectively. (C) homozygous mutation c.10233G > A of CUBN gene in patient 3 and his patients. (D) Position of the CUBN variants along the cubilin protein. (E) Conservation of L3096, R2636 and W3411 (blue rectangle showed) in CUBN gene among different species. Wt: wild type; hom: homozygous; het: heterozygous

*CUBN* gene biallelic pathogenic variants were identified by family trio WES (whole exome sequencing) of three children and their parents. Patient 1 carried compound heterozygous *CUBN* gene mutations c.9287 T > C (p.L3096P) and c.122 + 1G > A, which came from his father and mother respectively (shown in Fig. 3 A). The former was a missense mutation of *CUBN* gene, not seen in ExAC database (http://exac.broadinstitute.org) and gnomAD database (https://gnomad.broadinstitute.org). Poly-phen2, SIFT, Mutation Taster software revealed the scores were 0.813(possibly damaging), 0.003(damaging), and 1.000(disease causing) respectively. This missense mutation was regarded as possibly pathogenic (PM2, PM3, PP3, and PP4), the other splice-site mutation was predicted as “most probably affecting splicing” by Human Splicing Finder, and regarded as pathogenic (PVS1, PM2, and PP4) according to ACMG criteria.

Patient 2 carried compound heterozygous *CUBN* gene mutations c.9287 T > C (p.L3096P) and c.7906C > T (p.R2636*), which came from his father and mother respectively (shown in Fig. 3 B). The former was the same missense mutation as in patient 1. The other was a nonsense mutation leading to truncation of the protein. The frequency of this mutation was 0.00007 in ExAC and 9.339*10^− 5^ in gnomAD database. Mutation Taster, CADD and DANN software showed the mutation scores were 1.000(disease causing), 55(damaging), 0.998(damaging). The nonsense mutation was regarded as pathogenic (PVS1, PM2, and PP4) according to ACMG criteria.

Patient 3 carried homozygous *CUBN* gene mutations c.10233G > A (p. W3411*) (shown in Fig. 3 C). It was a nonsense mutation leading to cubulin truncation, not seen in ExAC but reported with frequency of 8.128*10^− 6^ in gnomAD database. Mutation Taster, CADD, DANN software revealed the mutation scores were 1.000(disease causing), 51(damaging), 0.993(damaging). This nonsense mutation was regarded as pathogenic (PVS1, PM2, and PP4) according to ACMG criteria.

**Fig. 4:**
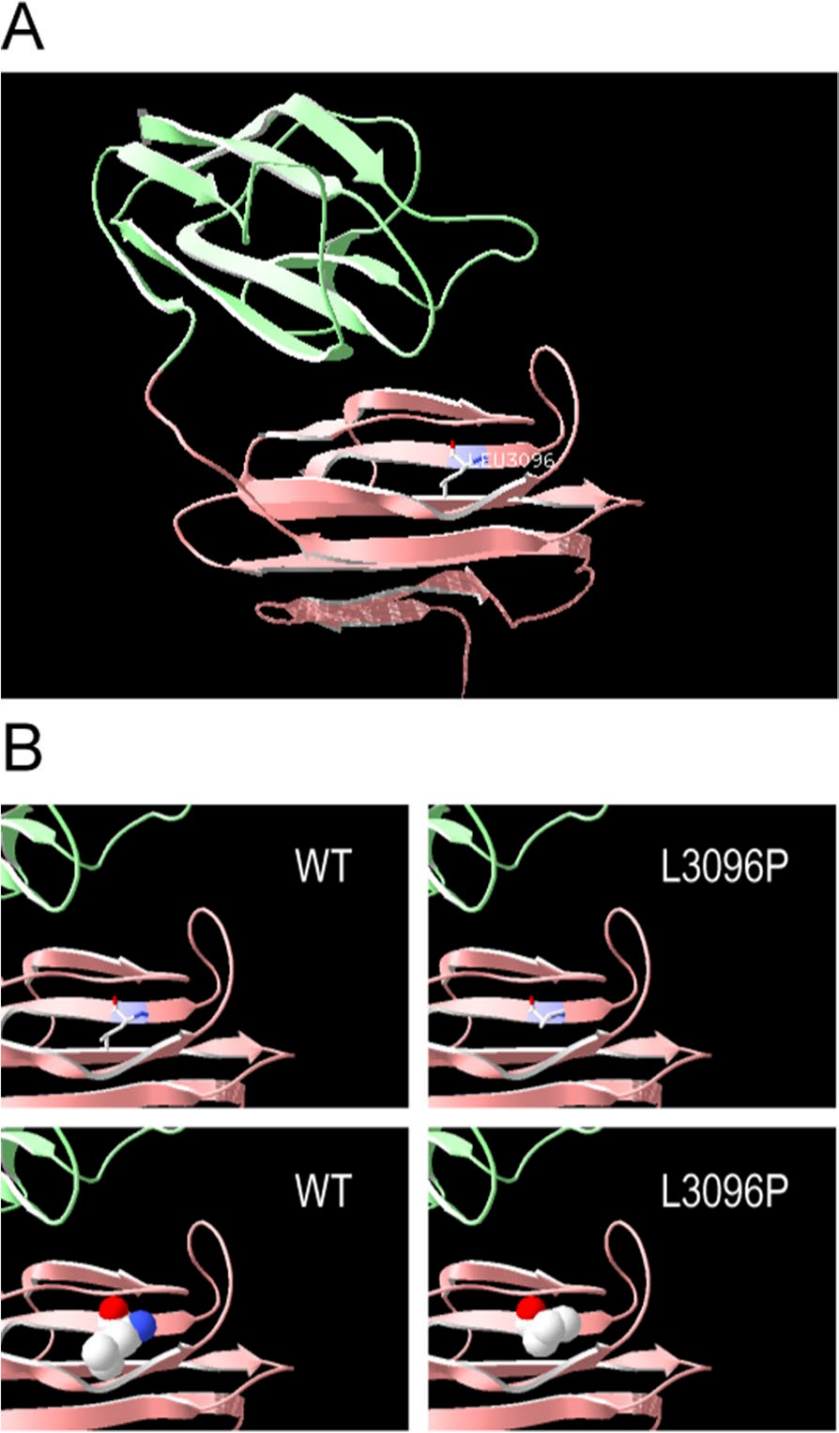
Protein modelling of CUBN variants. (A) CUBN are shown in ribbon format, colored green, pink, and purple, the purple indicates CUBN residue 3096. (B) The 3D-structure model and surface model between wild-type cubilin protein and variant cubilin protein

Except for the splicing mutation in patient 1, all other mutations were located at CUB domains 19, 23, 27 of cubilin (shown in Fig. 3 D). UCSC Genome Browser alignment indicated that L3096, R2636 and W3411 in *CUBN* gene was highly conserved among different species (shown in Fig. 3 E). Since two patients shared the same missense mutation (c.9287 T > C, p.L3096P), we built 3D-structure model of the mutant cubilin protein based on the solved wild-type CUBN 3D-structure by Swiss-Model (shown in Fig. 4 A). They share high sequence identity (6fzv, 36.25%). The 3D-structure model and surface model between wild-type cubilin protein and mutant cubilin protein were showed in Fig. 4 B. The hydrogen bond was calculated and found to be changed in the mutant compared with wild-type.

## Discussion

Previous studies have demonstrated that *CUBN* gene mutations can cause IGS that typically manifests as megaloblastic anemia and secondary neurological symptoms, with or without proteinuria. Isolated proteinuria caused by *CUBN* gene mutations is rare, only a few cases have been reported in the world so far. Boger et.al [18] found a missense mutation (I2984V) of *CUBN* gene which is associated with albuminuria in general population and in individuals with diabetes. In 2011, Ovunc et.al [3] identified a homozygous frameshift mutation in *CUBN* gene in two siblings of consanguineous patients with intermittent nephrotic-range proteinuria, which indicated that cubilin mutation may be considered as a rare single-gene cause of nephropathy. Recently Bedin et al. [14] identified 39 patients with biallelic *CUBN* gene mutations from three cohorts, and found that they had sub-nephrotic proteinuria and normal renal function. Our cases add more evidence that *CUBN* gene mutations may cause moderate proteinuria.

Cubilin works as a receptor for albumin, its defect may significantly reduce albumin reabsorption in renal proximal tubular cells and leads to the occurrence of proteinuria. Amsellem et.al [19] found that selective daily albumin excretion was increased approximately six-folds in cubilin-deficient mice. It is assumed that albuminuria come from renal tubular malabsorption in *CUBN* mutated patients, but whether it is also resulted from glomerular loss remains to be elucidated. Although there was no direct evidence to support glomerular origin, our cases showed pathological changes of podocytes and FSGS and their proteinuria gradually attenuated after administration of tacrolimus. Those suggest that albuminuria was probably not only originated from renal proximal tubule malabsorption, but may also from podocyte disfunction in *CUBN-*mutated patients.

Due to the scarcity of cases with *CUBN* gene mutations, kidney biopsy has been performed in only a few patients so far. Until now, focal segmental glomerulosclerosis has just been reported in 1 of 4 patients in genetic kidney disease cohort II with *CUBN* gene mutations [14]. In 2012, Prabakaran et.al [5] found cubilin expression in rat podocytes and human podocytes. Gianesello et.al [20] proved that cubilin mediates albumin endocytosis in human podocytes, *CUBN* gene mutations may lead to the dysfunction of cubilin, thus affect albumin endocytosis in podocytes. The development of proteinuria is usually related to podocyte damage, such as podocyte foot process effacement and cell loss [21]. Compared to megalin, cubilin is thought to have higher binding affinity for albumin [22]. Megalin can function as a sensor of albumin to determine the effect on cell survival. No or only a small amount of albumin binding to megalin can inhibit podocyte apoptosis, however, a large quantity of albumin binding to megalin can promote cell apoptosis [5]. It’s thought that the *CUBN* gene mutations lead to reduction of cubilin-binding ability with albumin, which make more free albumin available to bind with megalin. Consequently, it promotes podocyte apoptosis via the PI-3 K/PKB pathway, leading to a decreased number of podocytes. As a kind of terminal differentiated cell, podocyte can’t proliferate to compensate its loss, thereafter glomerular basement membrane become naked, followed by development of FSGS. Bedin et.al [14] reported the renal pathologies in 19 patients with *CUBN* gene mutations, most of them were minimal change disease or no lesions. But there were two patients whose lesions tended to be in the early stage of FSGS [14]. Interestingly, those pathologically manifested with FSGS unanimously had at least a relatively serious mutation in one allele, such as nonsense mutation, insertion, deletion or mutation in splice sites which led to frameshift or protein truncation. Since renal biopsy was rarely performed in patients with *CUBN* gene mutations, even less for electron microscopy (EM) examination. Therefore, the pathological changes of podocytes were not noted on EM in the past. The observation of obvious podocyte abnormalities with EM in our cases provide evidence for its involvement in the pathogenesis of albuminuria in *CUBN* gene-mutated patients.

Bedin et.al [14] firstly noted that all proteinuria-associated *CUBN* mutations were localized to C-terminal CUBN domains. Our patients showed that except for one intron splice-site mutation, other mutations are located in highly conserved sites at the C-terminal, CUB19, CUB23 and CUB27 domain respectively. All our cases had at least one relatively serious mutation and severe pathological changes accordingly.

In this study, four biallelic pathogenic variants of *CUBN* gene were identified in three isolated proteinuric children. The results demonstrate that *CUBN* gene mutations may cause isolated proteinuria pathologically presented as FSGS. Our cases extend the spectrum of renal manifestation and genotype of *CUBN* gene mutation.

## Data Availability

The data that support the finding of this study are available from the corresponding author upon reasonable request.

## Abbreviations

FSGS: Focal segmental glomerulosclerosis
CSA: Clinic Sequence Analyzer
ACMG: American College of Medical Genetics
IGS: Imerslund-Gräsbeck Syndrome
EM: Electron microscopy.
